# Redifferentiation of BRAF V600E-Mutated Radioiodine Refractory Metastatic Papillary Thyroid Cancer After Treatment With Dabrafenib and Trametinib

**DOI:** 10.7759/cureus.17488

**Published:** 2021-08-27

**Authors:** Sabih Jafri, Abid Yaqub

**Affiliations:** 1 Internal Medicine, University of Cincinnati, College of Medicine, Cincinnati, USA; 2 Endocrinology, University of Cincinnati, College of Medicine, Cincinnati, USA

**Keywords:** dabrafenib, trametinib, metastatic papillary thyroid cancer, redifferentiation, radioiodine, braf v600e mutation, mek inhibitor

## Abstract

Radioactive iodine-refractory metastatic differentiated thyroid cancer (RAIR) is associated with a poor prognosis. Multikinase inhibitors have demonstrated improvement in progression-free but not overall survival in such patients, but usage is limited by significant adverse effects and the development of resistance. Clinical research has demonstrated improvement in progression-free survival with the combined use of the BRAF/MEK inhibitor in patients with metastatic melanoma and anaplastic thyroid cancer with the BRAF^V600E ^mutation and has shown promise in redifferentiation of BRAF-positive RAIR differentiated thyroid cancer.

A 58-year-old woman went to her primary care physician for a growing mass on the left side of her neck. CT imaging noted a 6 x 8 x 6 cm mixed cystic and solid mass and lymphadenopathy. Core biopsy subsequently showed metastatic papillary thyroid cancer (Stage III, PT4a/PN1b), and she underwent a total thyroidectomy with left neck dissection. She then received 204mCi ^131^I post-total thyroidectomy. Unfortunately, her thyroglobulin continued to increase post-radioactive iodine (RAI) treatment, indicating persistent and/or recurrent thyroid cancer. An RAI-131 whole-body scan on the thyrogen protocol showed no significant RAI uptake. A fluorodeoxyglucose (FDG)-positron emission tomography (PET) CT scan was then performed, which showed recurrent metastatic disease with hypermetabolism noted in the left thyroid bed and FDG-avid bilateral cervical lymph nodes and pulmonary nodules. Given these findings, her cancer was classified as radioactive iodine refractory (RAIR). Molecular testing indicated the BRAF^V600E^ mutation. After a discussion with the patient, it was decided to initiate therapy with a BRAF inhibitor (dabrafenib 150 mg twice a day) and MEK inhibitor (trametinib 2 mg once a day) in an attempt to redifferentiate RAIR. Repeat RAI-131 thyrogen whole body scan one month after initiation of therapy demonstrated left level 2 cervical lymphadenopathy radioiodine uptake. The patient subsequently received 216 mCi ^131^I treatment given evidence of redifferentiation. Her post-treatment scan indicated additional uptake in a left lower lobe pulmonary nodule as well as a left paratracheal mass indicating successful RAI-131 uptake by metastases. Her thyroglobulin level, six months post-RAI, decreased to 4.0 indicating an encouraging response. Further surveillance, including imaging studies, is planned.

This case illustrates the re-differential potential for dabrafenib and trametinib treatment in patients with BRAF^V600E^-mutated RAIR differentiated thyroid cancer. This therapy has been shown to be successful in small series of patients and could potentially be offered to RAIR patients with the BRAF^V600E^ mutation as an alternative to multikinase treatment given its favorable side-effect profile.

## Introduction

An increasing incidence of thyroid cancer has been reported in the United States recently. The American Cancer Society notes an estimated 44,280 new cases and 2,200 deaths in 2021 [1]. The overall five-year survival rate hovers around 98% [1] though that number decreases significantly with metastatic disease. The standard of care therapy for metastatic differentiated thyroid cancer is radioactive iodine I^131 ^(RAI), which has been shown to reduce disease recurrence and mortality in high-risk patients. Disease refractory to RAI (noted as RAIR) portends a poor prognosis. The 10-year survival rate among patients with metastatic disease with noted ^131^I uptake is 56%, and it plummets to 10% in patients without any ^131^I uptake [2]. Genetic alterations, including RET-PTC translocations and BRAF^V600E^ and RAS point mutations, have been commonly identified in the molecular pathogenesis of papillary and follicular thyroid cancer besides other less common genomic alterations [3]. In addition, increased expression of vascular endothelial growth factor (VEGF) has been shown to play a role in assessing the aggressiveness of thyroid cancer [4].

The last decade has seen significant developments in treatment for advanced RAIR thyroid cancer. Multikinase inhibitors, including sorafenib and lenvatinib, have demonstrated improvement in progression-free but not overall survival in randomized clinical trials [5-6]. These agents have been Food and Drug Administration (FDA) approved for use in progressive RAIR patients. However, their usage is limited by significant adverse effects and the development of resistance.

Activation of mitogen-activated protein kinase (MAPK) has been shown to result in reduced expression of sodium/iodide symporter (NIS), which, in turn, leads to reduced iodide uptake, and, hence, resistance to the effects of RAI therapy [7]. BRAF and MEK inhibitors work to restore NIS expression by inhibiting the MAPK pathway [8].

Clinical trials have demonstrated improvement in progression-free survival with the combined use of BRAF/MEK inhibitor in patients with metastatic melanoma and anaplastic thyroid cancer with BRAF^V600E^ mutation resulting in FDA approval of these agents for these cancers [9-12].

Given the success of BRAF/MEK inhibitor combination therapy in the above-mentioned BRAF^V600E^-positive malignancies, this regimen has also been used in some small case series of BRAF^V600E^-positive RAIR thyroid cancer with promising results [13-15]. We present our experience in a patient with BRAF^V600E^-positive RAIR advanced papillary thyroid cancer who had a favorable response to a combined regimen of dabrafenib and trametinib therapy.

## Case presentation

In March 2019, a 58-year-old woman with a past medical history significant for hypertension and type 2 diabetes mellitus presented to her primary care physician for a growing mass on the left side of her neck. CT imaging of the neck was obtained, which depicted multiple mass lesions in the left neck, with the largest measuring 6 cm x 8 cm x 6 cm and characterized as a mixed cystic and solid mass displacing the trachea and other related structures rightwards. A core biopsy of the mass was then obtained. Epithelial cells were diffusely positive for HBME and vimentin, variable for CK903, and weakly for CD57 with a low Ki67 proliferation rate. These makers were suggestive of thyroid cancer and subsequent staging revealed metastatic papillary thyroid cancer (Stage III, PT4a/PN1b). She underwent a total thyroidectomy with left neck dissection. She was noted to have esophageal serosal, perineural, and angioinvasion involvement of the tumor per the operative report. Her postoperative course was complicated by perioral numbness thought to be secondary to hypocalcemia for which she received calcitriol and calcium carbonate with prompt resolution of her symptoms. She was then seen by endocrinology and underwent 204 mCi of ^131^I in July 2019. A ^131^I scan at the time of administration showed uptake in the anterior neck.

**Figure 1:**
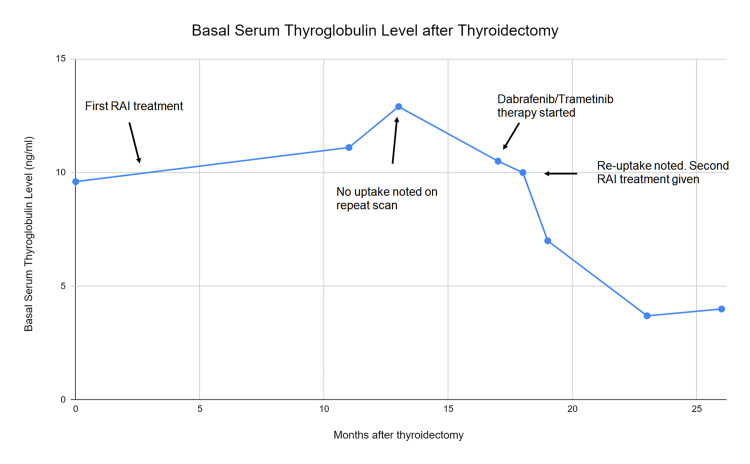
Trend of basal serum thyroglobulin levels in our patient post-thyroidectomy

**Figure 2:**
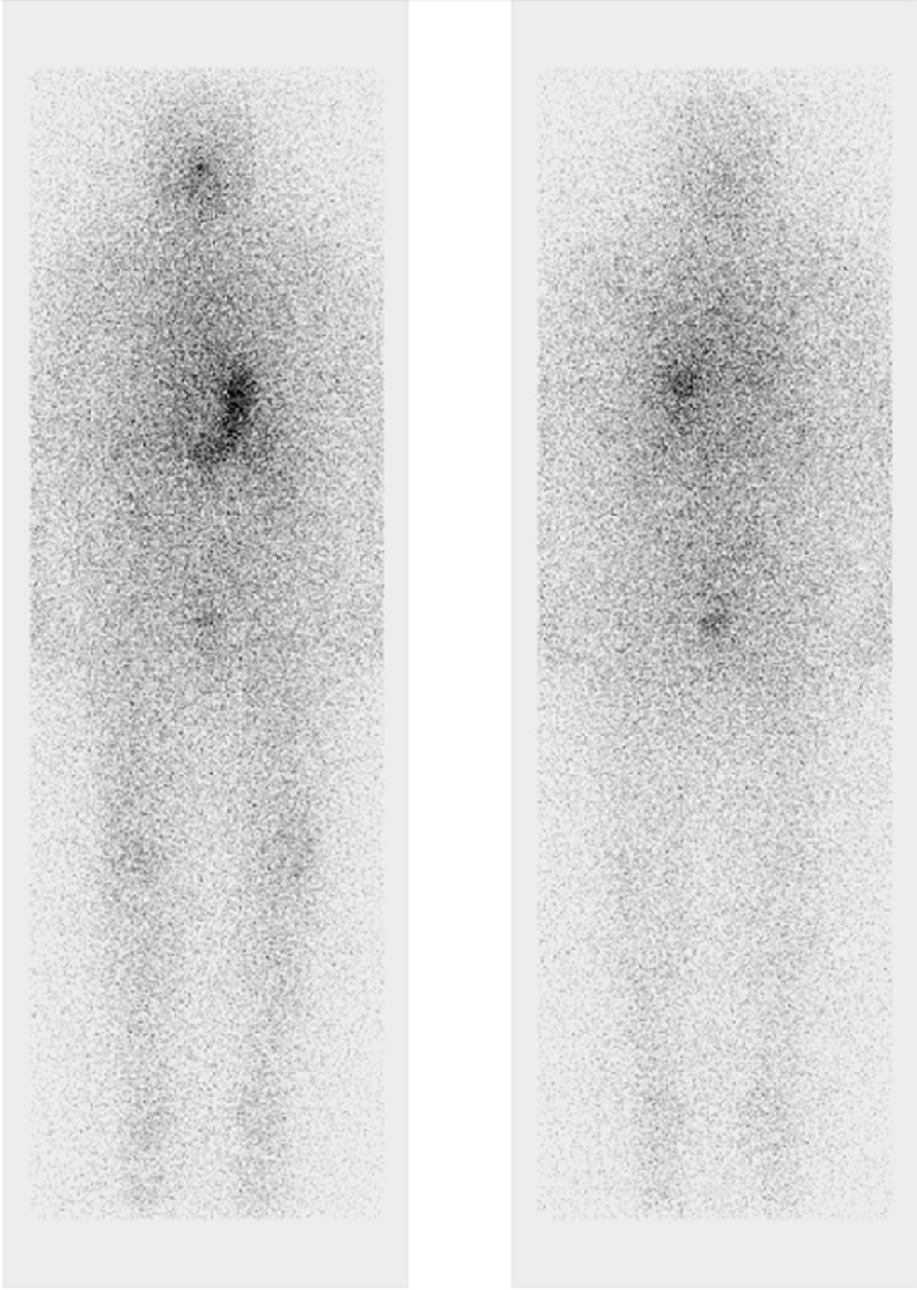
Anterior (left) and posterior (right) thyrogen-stimulated RAI-131 whole-body scan planar images showing no abnormal radioiodine uptake

**Figure 3:**
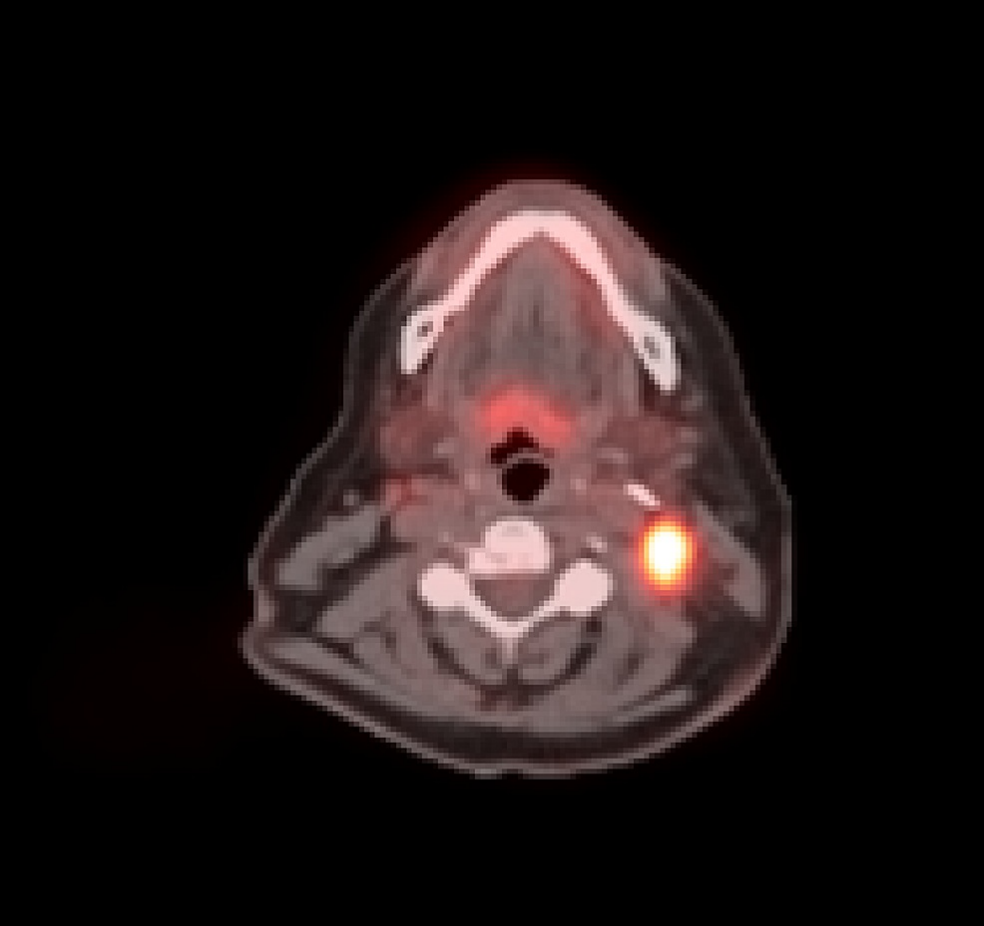
Hypermetabolic uptake noted in a left lateral cervical lymph node on positron emission tomography scan

In December 2019, her thyroglobulin was noted to be increasing (Figure 1), and she was scheduled for a repeat ^131^I treatment. Unfortunately, a ^131^I Thyrogen whole body scan prior to administration showed no uptake either in the thyroid bed or elsewhere in the body (Figure 2), so treatment was aborted. She then underwent a positron emission tomography in July 2020, which showed PET-avid metastatic disease in the thyroid bed and bilateral cervical lymph nodes (Figure 3). At that point, her disease was classified as refractory to RAI therapy, and a decision was made to start systemic therapy. Caris (molecular/genomic; Caris Life Sciences, Irving, TX) testing was obtained, which showed BRAF^V600E^ -mutation. She was started on dabrafenib (BRAF inhibitor) and trametinib (MEK inhibitor) in November 2020 in an attempt to redifferentiate RAIR. Adverse effects were self-limited and included mild nausea and rash.

**Figure 4:**
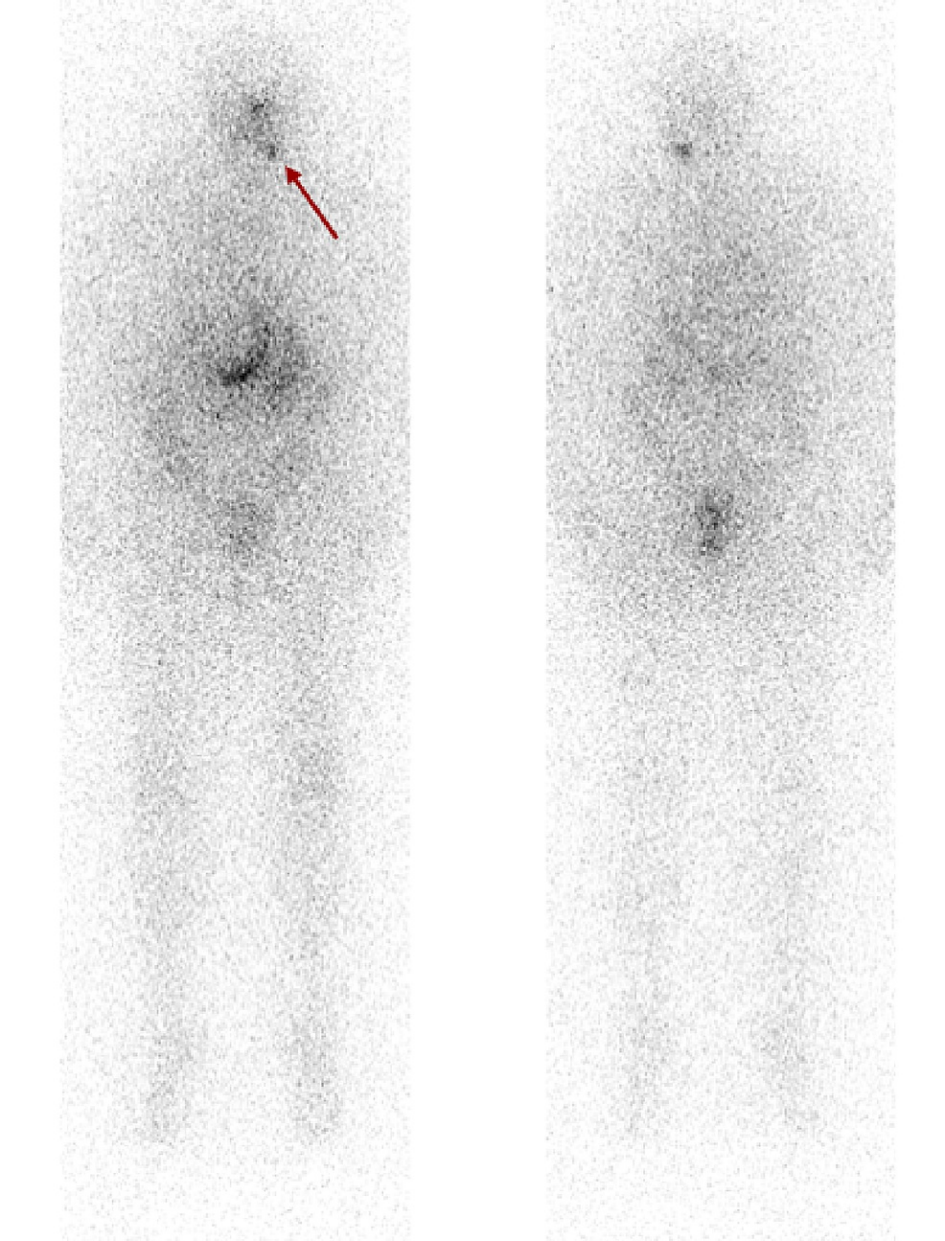
Anterior (left) and posterior (right) pre-therapy thyrogen-stimulated RAI-131 whole-body scan showing radioiodine uptake in the left cervical neck region BRAF/MEK inhibitor therapy (red arrow)

**Figure 5:**
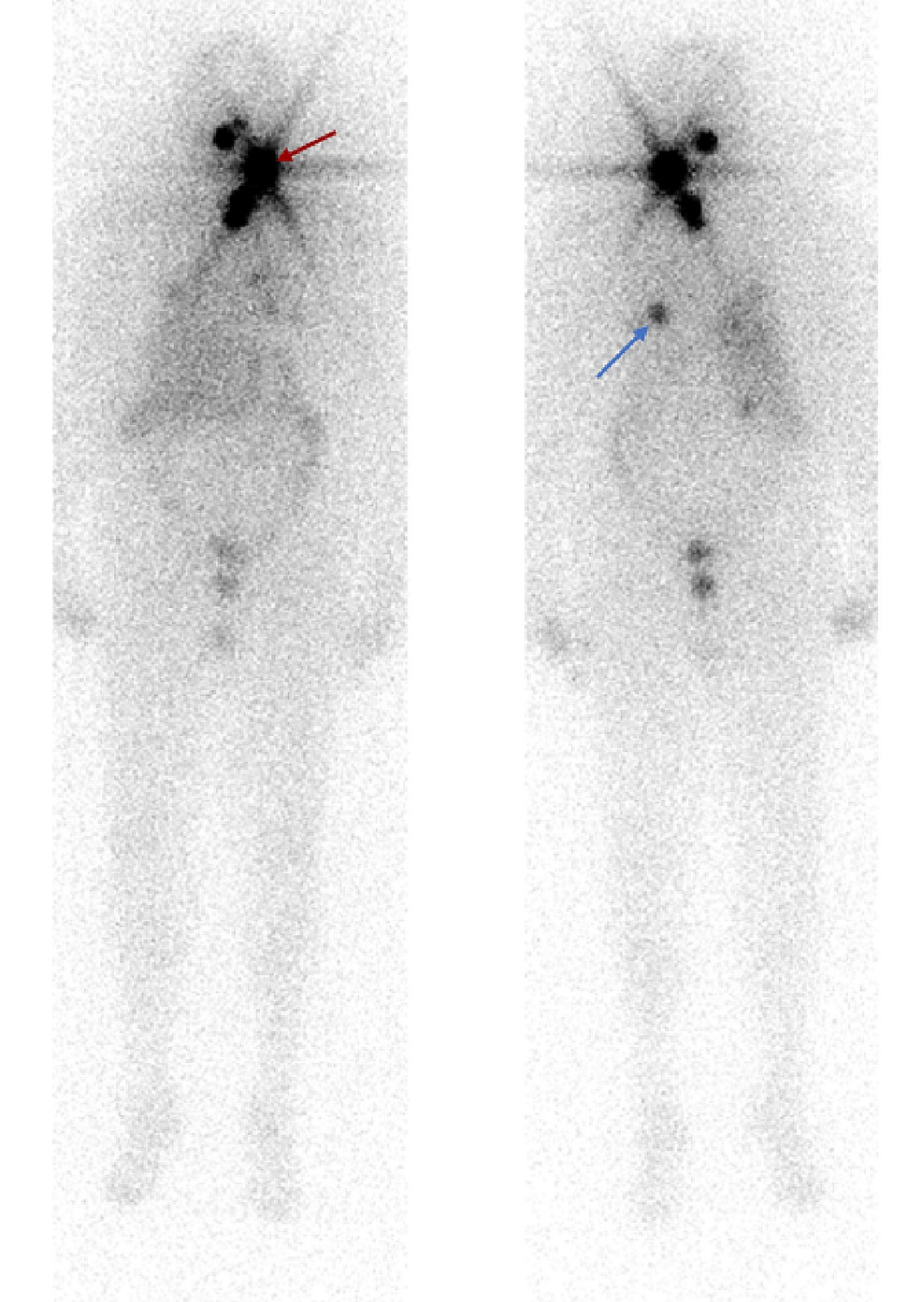
Anterior (left) and posterior (right) post-therapy thryogen-stimulated whole-body scan showing radioiodine uptake in the left cervical neck region (red arrow) and left lower lobe pulmonary nodule (blue arrow) following BRAF/MEK inhibitor redifferentiation therapy

She subsequently underwent a ^131^I whole-body scan on the thyrogen protocol after four weeks of combined dabrafenib/trametinib therapy. The pre-therapy scan showed radioiodine uptake in a left level-2 cervical lymph node and a soft tissue nodule in the left lower neck (Figure 4). Given encouraging signs of radioiodine uptake, she underwent ^131^I treatment with 216 mCi in late December 2020. The post-therapy scan showed radioiodine uptake within the left level 2 cervical lymph node, left paratracheal soft tissue, as well as a now-demonstrated 7 mm left lower lobe nodule (Figure 5). Dabrafenib and trametinib were held after one week of RAI-131. Her labs in June 2021 showed thyroglobulin, which had decreased to 4.0 ng/ml from a basal level ~10ng/ml and a stimulated level of 1,143 ng/ml (Figure 1). She continues to be under the surveillance phase with intermittent lab monitoring.

## Discussion

Due to the increased morbidity and mortality associated with RAI-refractory thyroid cancers, there is an ongoing need for the development of agents that can show effectiveness despite RAI refractoriness. The last decade has seen considerable progress in broadening our understanding of molecular mechanisms underlying thyroid carcinogenesis and RAI-refractoriness. Such research has paved way for the development of agents specifically targeting the driver mutations that play an instrumental role in RAI refractoriness.

Two phase III trials have shown the benefits of using multikinase inhibitors to treat RAIR metastatic thyroid cancer: sorafenib in the DECISION trial [5] and lenvatinib in the SELECT trial [6], both showed increased median progression-free survival compared to placebo. However, improvement in overall survival was not seen in either of these trials. These studies formed the basis for FDA approval of these agents for the treatment of advanced RAIR patients. These newer systemic agents have since been added to the National Comprehensive Cancer Network guidelines for the treatment of thyroid cancer [16].

Unfortunately, treatment-related significant adverse effects are quite common with these agents, leading to dose reduction and temporary or permanent discontinuation in many patients, which can potentially decrease long-term utilization.

There has been a growing interest in the development of targeted therapies for thyroid cancer-directed against specific genomic alterations. The most common BRAF mutation noted in papillary thyroid cancer is BRAF^V600E^. It is associated with increased incidence of lymph node metastasis, extra-thyroidal extension, and increased recurrence [17].

Activation of mitogen-activated protein kinase (MAPK) has been shown to result in reduced expression of sodium/iodide symporter (NIS), which, in turn, leads to reduced iodide uptake [7]. BRAF and MEK inhibitors work to restore NIS expression by inhibiting MAPK pathway activation [8]. BRAF and MEK inhibitors have been shown in clinical trials to be effective for the treatment of BRAF^V600E^-mutated melanoma, and, more recently, BRAF^V600E^-mutated anaplastic thyroid cancer and are now FDA approved for the treatment of these malignancies [9-12].

Due to the demonstrated success of BRAF and MEK inhibitors in BRAF-mutated melanoma patients, their use was studied in well-differentiated thyroid cancer patients due to the potential for the redifferentiation and restoration of RAI-uptake. Ho et al. [18] demonstrated that selumetinib, a MEK inhibitor, increased the uptake of iodine-124 in patients with RAIR papillary thyroid cancer by inhibiting the MAPK pathway. This effect was observed to be particularly effective in patients with RAS-mutant tumors. In addition, small phase I and II clinical trials have shown that BRAF inhibitors, dabrafenib and vemurafenib, can stimulate RAI-uptake in BRAF^V600E^-positive RAIR papillary thyroid cancer [13-15]. Rothenburg et. al [13], for example, demonstrated enhanced radioiodine uptake with dabrafenib in 6/10 patients with BRAF-mutant papillary thyroid cancer while Irvani et al. [15] noted the restoration of RAI uptake with the use of combined BRAF and MEK inhibition in all patients with BRAF^V600E^ mutation.

We present here a case of a patient who experienced the redifferentiation of her previously RAI-refractory metastatic papillary thyroid cancer after treatment with dabrafenib and trametinib. Our patient demonstrated significantly increased RAI-131 uptake in metastatic lesions, which previously did not show any RAI uptake, suggesting redifferentiation. This observation, combined with findings from other clinical case series in the above-mentioned series, suggests that these agents can be a potential therapeutic option for patients with BRAF^V600E^-mutated metastatic papillary thyroid cancer who are not candidates for or are unable to tolerate adverse effects of multikinase inhibitor therapy. In addition, successful redifferentiation followed by RAI-131 therapy could prevent the need for long-term systemic treatment, hence minimizing significant adverse effects and improving the quality of life for patients. This regimen was well-tolerated with grade I adverse effects (nausea, rash) in our patient. It is unknown currently whether this combination therapy with BRAF/MEK inhibitors results in an improvement in the progression-free or overall survival of patients. A multicenter randomized phase II trial in papillary thyroid cancer patients is currently underway to address this question (NCT01723202).

## Conclusions

Metastatic RAIR thyroid cancer is associated with a poor prognosis. Recently, significant research efforts have been directed to develop therapeutic agents that can induce redifferentiation and hence reverse RAI-refractoriness in such patients. Our patient described in this case report as well as other patients in the small clinical case series described here have demonstrated encouraging results in patients treated with a combination of BRAF and MEK inhibitors. Multicenter randomized control trials are needed to better elucidate treatment outcomes with the use of these agents.
